# One Novel 2.43Kb Deletion and One Single Nucleotide Mutation of the *INSR* Gene in a Chinese Neonate with Rabson-Mendenhall Syndrome

**DOI:** 10.4274/jcrpe.5080

**Published:** 2018-05-18

**Authors:** Xiang Chen, Huijun Wang, Bingbing Wu, Xinran Dong, Bo Liu, Hongbo Chen, Yulan Lu, Wenhao Zhou, Lin Yang

**Affiliations:** 1 Children’s Hospital of Fudan University, Clinic of Neonatology, Shanghai, China; 2 Children’s Hospital of Fudan University, Key Laboratory of Birth Defects, Shanghai, China; 3 Children’s Hospital of Fudan University, Key Laboratory of Neonatal Diseases, Shanghai, China; 4 Children’s Hospital of Fudan University, Clinic of Endocrinology, Genetics and Metabolic Diseases, Shanghai, China

**Keywords:** Insulin receptor gene, Rabson-Mendenhall syndrome, neonate, mutation, next generation sequencing

## Abstract

Mutations in the insulin receptor (*INSR*) gene are responsible for Donohue syndrome (DS) and Rabson-Mendenhall syndrome (RMS). Insulin resistance is a feature of both diseases.

Our patient was a Chinese neonate suffering from abnormal glucose homeostasis, hyperinsulinemia, dry skin, heavy hair, growth retardation and an elevated testosterone level. To search for candidate point mutations, small insertions or deletions and copy number variants, 2742 inherited disease-gene panel sequencing was performed. One pathogenic mutation (c.3355C>T, p.Arg1119Trp) and a novel 2.43Kb deletion (chr19:7150507-7152938) in INSR were found. The patient was diagnosed as RMS. Sanger sequencing and real-time quantitative polymerase chain reaction (PCR) confirmed the missense variant and microdeletion, respectively. We therefore supposed that these variants were candidate mutations in this case. We report a novel 2.43Kb deletion in INSR gene and provide further proof of the power of next generation sequencing in rare disease diagnosis.

## What is already known on this topic?

Mutation of the insulin receptor gene is responsible for Rabson-Mendenhall syndrome (RMS) which is an autosomal recessive disorder. Typical symptoms of RMS include growth retardation, elfin face, gingival hyperplasia, acanthosis nigricans, hypertrichosis and insulin resistance.

## What this study adds?

We report an atypical and mild RMS patient due to a compound heterozygosity consisting of a novel 2.43Kb deletion and a known, pathogenic point mutation in the *INSR* gene.

## Introduction

Insulin receptor *(INSR)* is the gene responsible for a series of insulin resistance diseases, including hyperinsulinemic hypoglycemia, familial 5 [Online Mendelian Inheritance in Man (OMIM)#609968], Donohue syndrome [(DS), also called leprechaunism; OMIM#246200] and Rabson-Mendenhall syndrome [(RMS); OMIM#262190]. The inheritance pattern of DS and RMS is autosomal recessive. Typical symptoms of DS include growth retardation, elfin face, gingival hyperplasia, acanthosis nigricans, hypertrichosis and insulin resistance (1). RMS and DS share similar symptoms. The symptoms of DS are more severe, have an infantile onset and may lead to early death. RMS is often encountered as being of childhood-onset and with survival up to adulthood with milder symptoms. The differential diagnosis is based on the onset age and severity of the disease (2).

In this study, we describe a Chinese, male, newborn with hyperinsulinemia and hyperglycemia. Next generation sequencing (NGS) detected a compound heterozygous mutations of INSR, including one known mutation and one novel 2.43Kb deletion.

## Case Report

The proband is a male infant and the first child of non-consanguineous parents. During the fetal period, he was diagnosed with intrauterine growth retardation and oligohydramnios and was delivered by natural labor at 36 weeks of gestation with a low birth weight at 1.7 kg. At age 13 days, he presented with dry skin and heavy hair over his whole body. The plantar grasp, Moro and sucking reflexes were weak. Facial malformation, abnormality of mouth size, acanthosis nigricans and abdominal distention were not observed. Clinical tests showed hyperglycemia (14.7 mmol/L), hyperinsulinemia (>300 IU/mL) and fasting hypoglycemia. C-peptide was 4.05 ng/mL (1.10-4.40 ng/mL). HbAlc (glycosylated hemoglobin) was normal (5.4%). Other abnormal laboratory test results are shown in Table 1. Insulin auto-antibodies were negative. Routine blood tests, liver function tests and thyroid-stimulating hormone levels were normal. Echocardiography suggested a ventricular septal defect, an atrial septal defect and ultrasonography indicated swelling of both kidneys. Magnetic resonance imaging of the brain indicated an impaired myelination of white matter. The mother had transient hypothyroidism during pregnancy. Otherwise the family history is negative. At the last outpatient follow-up at age four months, the patient’s neurodevelopment was found to be delayed and that the high postprandial blod glucose (>11 mmol/L) and hyperinsulinemia (>300 IU/mL) persisted.

Pre-test counseling was performed by physicians and appropriate informed consent was signed by the patient’s parents in the clinic. The criteria of genetic testing received approval from the ethics committees of the Children’s Hospital, Fudan University (2016-235). Genomic DNA samples were extracted from whole blood using the QIAamp DNA Blood Mini kit (QIAGEN, Germany) following the manufacturer’s protocol. The quality and quantity of the DNA samples were measured using a NanoDrop 2000 spectrophotometer (Thermo Fisher Scientific, USA). Nucleic acid preparation and high-throughput sequencing were performed using standard protocols in a Clinical Laboratory Improvement Amendments (CLIA) compliant sequencing laboratory in Wuxi NEXTCODE (288 Fute Zhong Road, Waigaoqiao Free Trade Zone Shanghai 200131, China CLIA ID 99D2064856). Inherited-Disease panel sequencing was generated using the Agilent ClearSeq Inherited Disease kit, Illumina Cluster and SBS kit. The sequencing was performed using NGS on the Illumina Hiseq 2000/2500 platform. This covers a minimum of 98% of the genome with 20X coverage and was compared to a human reference sequence.

**Figure 1:**
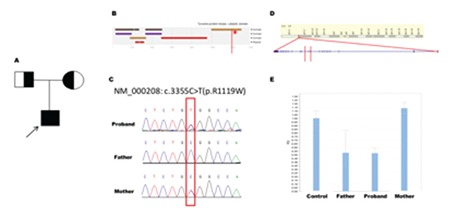
Insulin receptor gene compound heterozygous mutation: a known missense mutation and a novel microdeletion. A) Pedigree of the family. B) The SNV is located at the tyrosine-protein kinase catalytic domain and marked by red asterisk. C) Sanger sequencing shows the mutation is from mother. Insulin receptor gene locates at 19p13.3-19p13.2. D) The deletion fragment is marked within two red lines. This fragment contains Exon11 and part of Exon 10. E) Real-time quantitative polymerase chain reaction shows that the deletion is from father

We identified a known-pathogenic mutation (c.3355C>T, p.Arg1119Trp) at exon 18 of the *INSR* gene (NM_000208). This mutation has been reported in a patient with DS (3). This mutation was recorded in HGMD (http://www.hgmd.cf.ac.uk/ac/index.php) as CM1119. The Exac database (http://exac.broadinstitute.org/) and the 1000 gene database (http://www.internationalgenome.org/) have no record of this variant. This mutation was also the only record in our internal database, which contains sequencing data of ClearSeq of 4071 patients. Paired primers were designed by using Primer3 website and primer-BLAST (5’-GGGAGGAGAACCCTGGTGAG-3’ and ’-ATCCGAGGAGGCCAGGAG-3’). Sanger sequencing indicated that this mutation was inherited from the mother (Figure 1A, 1B, 1C).

**Figure 2:**
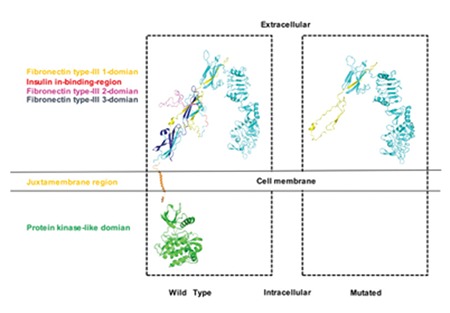
3D structural modeling of insulin receptor protein. Comparing to the wild type, 3D structural modeling estimates a large portion of deficiency in the monomer form of the insulin receptor caused by the deletion. Different domains are marked by different colors with the color of the domain matched by the color of the domain name in the key

We used CANOES (CNVs with an Arbitrary Number of Exome Samples) for basic detection of CNVs from NGS data at gene-level and region-level (4). Gene-level annotation was based on OMIM, Human Gene Mutation Database (HGMD), Swiss-Prot and RefSeq. Region-level information was annotated by Database of Genomic Variants (DGV), and Database of Genomic Variation and Phenotype in Humans using Ensembl Resources (DECIPHER). We detected a novel deletion of approximately 2.43 Kb (chr19:7150507-7152938) within the *INSR* gene (Figure 1D). This deletion is not found in HGMD, DECIPHER or DGV. Additionally, it is absent from our internal database. The detected mutation was confirmed using real-time quantitative PCR. PCR-amplified DNA products were subjected to direct automated sequencing (ABI step one plus v.2.0). Both strands of each amplicon were sequenced using the primers 5’-CCTGACCTGGGGACGAAAA-3’ and 5’-GTCTCCACCATTCGAGTCTGA-3’. Real-time quantitative PCR indicated the deletion was from the father (Figure 1E). This region covers part of exon 10 and all of exon 11. The deletion is estimated to cause a truncated protein. We performed a three-dimensional (3D) structural modeling of the monomer form of the INSR and mapped the deletion on to it. The PBD number of the INSR extracellular region is 4ZXB.E, that of the juxtamembrane region is 2MFR.A and that of the tyrosine kinase domain is 3BU3.A (5). The 3D structural modeling shows the monomer form of the INSR. The Fibronectin type-III 2-domain, Fibronectin type-III 3-domain, Insulin in-binding-region, protein kinase-like domain, juxtamembrane region and partial Fibronectin type-III 1-domain are absent (Figure 2) (5).

## Discussion

In this Chinese newborn baby, we identified a novel microdeletion and a known missense mutation within the *INSR* gene, which caused a compound heterozygous mutation. The *INSR* gene is located at chromosome 19 and encodes the INSR. HGMD contains 178 mutations of *INSR*. For RMS, 26 mutations of INSR are reported. Of these, two compound heterozygous mutations, each containing one deletion and one single nucleotide mutation, have been previously reported. A gross deletion containing exons 9 and 10 was reported in a 15-year-old RMS patient (6). This patient carried a mutation (p.Ser635Leu) in INSR with a compound heterozygous genotype. The main phenotypes are hyperglycemia and hyperinsulinemia. Another gross deletion contains exon 18. This RMS patient, as a compound heterozygote, also carried a mutation (p.Val66Ala). Nephrocalcinosis was found to be one of the patient’s dominant features (7). For the patient we report, the missense mutation (c.3355C>T, p.R1119W) has been reported from a DS patient who had symptoms at birth and died at three months of age (3). Our patient had symptoms 13 days after birth. Some typical RMS features of this patient include abnormal glucose homeostasis, hyperinsulinemia, dry skin, thick hair, elevated testosterone and growth retardation. We diagnosed this patient as RMS. With a microdeletion, our patient presented a mild and atypical phenotype, which may be explained by the unclear genotype-phenotype correlation of mutations in INSR. Different missense mutations in the same codon relate to different phenotypes (8). One DS patient, bearing a homozygous deletion in INSR resulting in inactivation of the INSR lived for 3.5 years (9). The coexistence of modifier genes and compensatory pathways may explain the phenotypic variability (10).

The insulin receptor is a tetramer of two α monomers and two β monomers. It is widely expressed and plays a vital role as a mediator between the extracellular and intracellular insulin signaling pathway. The whole region of the α-subunit is extracellular. The α-subunit contains a Leu-rich-compositionally biased region, Cys-rich-compositionally biased region, a Fibronectin type-III 1-domain and an insulin in-binding-region. The β-subunit extends through the cell membrane into the cytoplasm. The extracellular region of the β-subunit contains a Fibronectin type-III 2-domain and a Fibronectin type-III 3-domain. The cytoplasmic region of the β-subunit consists of several functional domains including a juxtamembrane region, a tyrosine kinase domain and the carboxy-terminal-region (5,11). The α-subunit is responsible for binding affinity to insulin. The Cys-rich-compositionally biased region is the main binding site of insulin. Fibronectin type-III domains 1 and 2 form the secondary insulin-binding site (5). Deficiency of the juxtamembrane region makes the folding of IR unstable and affects its downstream processing (12). The function of the Fibronectin type-III 3-domain remains unknown. A helical transmembrane region follows the Fibronectin type-III 3-domain. Through binding with insulin, IR initiates the phosphorylation of different phosphotyrosine residues in the tyrosine kinase domain (13). Downstream IR substrates bind to phosphotyrosine residues of IR and regulate two main signaling pathways: the phosphatidylinositol 3-kinase-AKT/protein kinase B (PI3K-AKT/PKB) pathway and the Ras-MAPK pathway. The PI3K-AKT/PKB pathway is responsible for controlling cell growth and differentiation. The metabolic action of insulin is mainly regulated by the Ras-MAPK pathway (14). 3D structural modeling indicates that with deficiencies in both α-subunit and β-subunit, IR may be unable to combine with insulin receptor substrates and recruit the downstream signaling molecules (15).

Our patient did not show some of the typical features of RMS including coarse face, gingival hyperplasia and acanthosis nigricans. These three symptoms can be absent in neonates and may develop in adolescence (16). The insulin receptor is expressed in the heart and regulates cardiac cell activity though the PI3K-AKT pathway. Insulin receptor deficiency possibly leads to cardiac dysfunction, as observed in some patients (10,17) and proven using animal models (18). There is no evidence indicating any relationship between heart structural malformation and insulin receptors, so we consider that the ventricle septal defect is not a consequence of insulin receptor deficiency in this case. The long-term prognosis of RMS patients is poor (19). Recombinant human insulin-like growth factor 1 and recombinant leptin are recommended for treatment of severe insulin resistance syndrome (19,20). However, the complications and safety of these drugs remain unknown (21).

In summary, we show an RMS patient carrying one known pathogenic mutation and one novel deletion in INSR. Since the presenting clinical features of patients with insulin resistance syndrome can be atypical, when the diagnosis is in doubt genetic testing may help to identify the final diagnosis.

## Figures and Tables

**Table 1:**
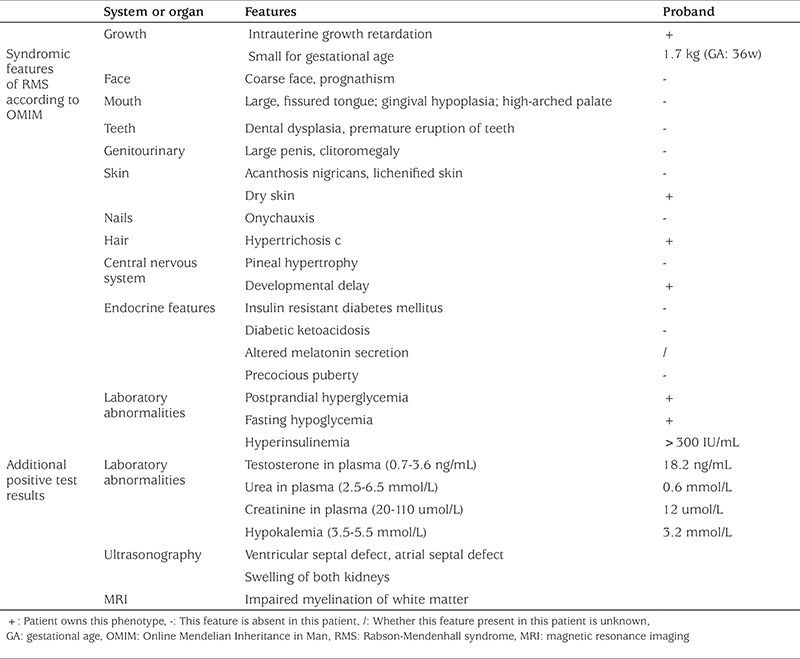
Clinical features of patient
